# A non-coding ABO regulatory variant associatedwith VWF levels, thrombosis risk, and COVID-19 severity is topologically linked to ADAMTS13 in endothelial cells

**DOI:** 10.1016/j.xhgg.2025.100550

**Published:** 2025-11-27

**Authors:** Douglas Victorino Esposito, Hellen Ferreira de Souza Sobrinho, Marcelo Rocha Marques

**Affiliations:** 1 Oral Biology Program, Piracicaba Dental School, University of Campinas, Piracicaba, São Paulo, Brazil; 2 Department of Biosciences, Piracicaba Dental School, University of Campinas, Piracicaba, São Paulo, Brazil

**Keywords:** thrombosis, von Willebrand Factor, ABO, ADAMTS13, COVID-19, endothelial cells, cis-regulatory element, CRISPRa, luciferase, genetic variants

## Abstract

Venous thromboembolism (VTE) is a major cause of mortality, influenced by genetic and environmental factors. von Willebrand factor (VWF) mediates hemostasis by promoting platelet adhesion, and its plasma levels are associated with thrombotic risk. Although many non-coding variants in *ABO* are associated with VWF levels, VTE risk, and COVID-19 severity, the mechanisms underlying these associations remain unclear. In this study, we identified the *ABO* locus as the genomic region with the highest concentration of variants associated with VWF levels. Chromatin conformation analyses in endothelial cells revealed non-coding *ABO* variants (rs657152, rs9411377, rs660340, and rs505922) associated with VWF levels, VTE risk, and COVID-19 severity, located in spatial proximity to *ADAMTS13*. ADAMTS13 is a key regulator of VWF activity, and both ADAMTS13 and VWF play crucial roles in coagulation and thrombosis. Chromatin activation (CRISPRa) of the region near the non-coding *ABO* variant rs657152 increased *ADAMTS13* transcription in endothelial cells, suggesting that this variant resides in a regulatory region with the potential to modulate long-range transcriptional control of *ADAMTS13*. Luciferase assay revealed reduced transcriptional activity driven by the rs505922-C allele in endothelial cells. These findings provide insights into the spatial organization of the *ABO* locus and its potential role in *ADAMTS13* regulation.

## Teaser

Non-coding *ABO* variants associated with thrombosis, von Willebrand factor levels, and COVID-19 severity reveal topological proximity to *ADAMTS13* in endothelial cells. Chromatin interaction data, CRISPRa assays, and enhancer activity analyses support a regulatory role for these variants in *ADAMTS13* expression, revealing a potential *cis*-regulatory mechanism contributing to coagulation phenotypes.

## Introduction

Thrombosis accounts for approximately one-fourth of global mortality, with venous thromboembolism (VTE [MIM: 188050]) encompassing deep vein thrombosis (MIM: 188050), and pulmonary embolism (MIM: 188050), representing a substantial contributor to this burden.^1^ VTE arises from a multifactorial interaction of genetic and environmental risk factors, with immobility, prolonged hospitalization, and malignancy-induced prothrombotic states being dominant contributors.^2^^,^^3^ Comorbidities such as obesity, advanced age, trauma, surgical procedures, renal insufficiency, autoimmune disorders, and COVID-19 highlight the complexity of VTE etiology.^4^^,^^5^^,^^6^

**Figure 1:**
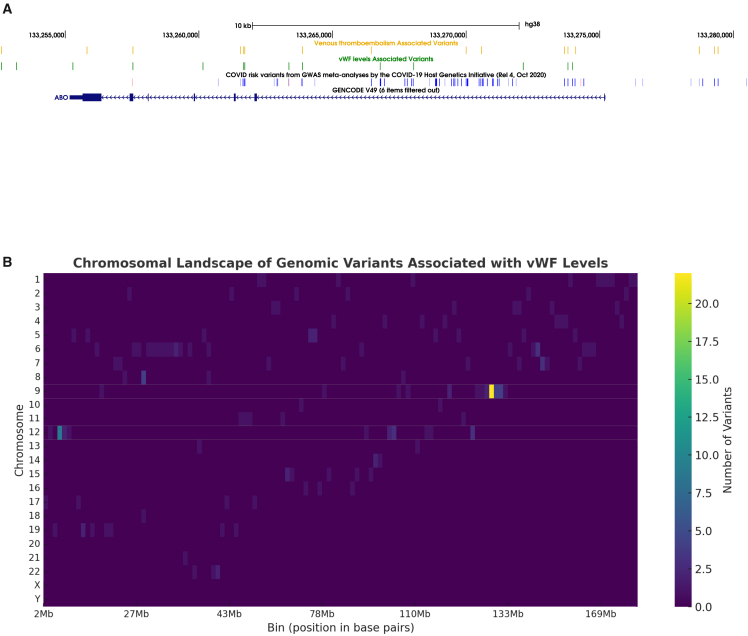
Variant mapping at the ABO locus (A) Genomic locus for *ABO*, highlighting non-coding variants within and near *ABO*. Variants are labeled according to their association with VTE, VWF levels, and COVID-19. The COVID-19 track shows variants with beta values (effect size) above the median in blue and below the median in lighter tones. (B) The heatmap displays the distribution of genomic variants associated with VWF levels across all chromosomes, including chromosomes X and Y. Data are binned into 100-kb intervals, with color intensity representing the number of variants within each bin. The region with the highest density of variants, located on chromosome 9, coincides with the *ABO* locus. The second region with the highest concentration of variants is located on chromosome 12, overlapping the VWF gene itself. These two enriched regions are highlighted on the respective chromosome tracks representing variant density. Chromosomes without detected variants in certain regions are represented by empty bins.

Many genetic VTE risk factors have been identified in loci directly involved in coagulation, including *F2* (MIM: 176930), *F5* (MIM: 612309), *F11* (MIM: 264900), *FGG* (MIM: 134850), *ABO* (MIM: 110300), *SERPINC1* (MIM: 107300), *PROCR* (MIM: 660646), *PROC* (MIM: 612283), and *PROS1* (MIM: 176880), providing insights into the genetic basis of its coagulation cascade.^7^ Non-coding variants within the *ABO* are associated with von Willebrand factor (VWF [MIM: 613160]) levels and VTE risk.^4^^,^^7^^,^^8^^,^^9^^,^^10^^,^^11^^,^^12^^,^^13^^,^^14^^,^^15^ VWF, a multimeric glycoprotein synthesized by endothelial cells and megakaryocytes, regulates hemostasis and thrombosis, with elevated plasma levels linked to VTE risk and lower levels to hemorrhagic disorders.^8^^,^^16^ Additionally, non-coding variants within the *ABO* locus have been reported to be associated with COVID-19 severity, VWF levels, VTE risk, and a spectrum of hematological, cardiovascular, and metabolic traits, in some cases across distinct studies and phenotypic analyses (Figure 1A; Table S1). However, the mechanisms through which these variants influence such diverse phenotypes remain only partially understood.

The ABO histo-blood group system is a major genetic determinant of circulating VWF levels^17^^,^^18^ through its influence on VWF glycosylation patterns,^19^ which modulate susceptibility to proteolytic cleavage and hepatic clearance. However, ABO-related mechanisms do not fully explain the marked inter-individual variability in VWF plasma concentrations.^20^

The association between non-coding variants in *ABO* and VWF levels, particularly in light of the incomplete explanatory power of glycosylation-mediated mechanisms, raises the possibility that these variants exert regulatory functions if functionally active. Since gene regulation often depends on the three-dimensional (3D) organization of chromatin, it is essential to determine whether these variants are positioned within structural domains that enable regulatory interactions with coagulation-related genes. To explore this, we investigated the topological organization of these variants and their spatial proximity to key coagulation genes in endothelial cells, providing insights into their potential functional relevance.

## Material and methods

### *In silico* analyses

To investigate the potential regulatory role of non-coding variants within the *ABO* locus, a manual inspection of the genomic neighborhood (±500 kb) of *ABO* was performed in search of biologically relevant candidate genes associated with VWF levels and thrombotic disorders, which led us to identify *ADAMTS13* (MIM: 604134). We then manually performed an integrative set of *in silico* analyses focused on endothelial cells.

#### Genome Browser visualization of genetic variants

Genetic variants associated with VWF levels, VTE, and COVID-19 were retrieved from the GWAS Catalog and visualized using the UCSC Genome Browser.

#### Heatmap analysis

To explore the genomic distribution of genome-wide association study (GWAS) variants associated with VWF levels, a heatmap was generated to visualize their positions across the genome. This analysis highlighted the enrichment of variants in specific chromosomal regions, aiding the identification of key regulatory loci and informing subsequent analyses.

#### Chromatin interaction data and analysis

Given their abundance and recognized contribution to circulating ADAMTS13, endothelial cells were used as models to investigate the 3D genome organization and regulatory interactions between the *ABO* and *ADAMTS13* loci through analysis of high-resolution chromatin conformation and epigenomic datasets, supported by extensive public resources.

##### Hi-C data

Chromatin conformation capture data from human umbilical vein endothelial cells (HUVECs), obtained from the 4D Nucleome Project (ID: 4DNESHFBC56P), were analyzed to map chromatin interactions of the *ABO* and *ADAMTS13* loci.

##### ChIA-PET data

Chromatin interaction analysis by paired-end tag sequencing (ChIA-PET) data for CTCF binding sites in HUVECs were retrieved from the ENCODE Project (ID: ENCSR404FPI-ENCFF446KBC).

##### DNase-seq data

DNase I hypersensitivity sequencing (DNase-seq) data for HUVECs, from ENCODE (ID: ENCSR000EKF-ENCFF671RMS), were analyzed to identify regions of accessible chromatin.

##### ChIP-seq data

Chromatin immunoprecipitation sequencing (ChIP-seq) datasets for RAD21, a cohesin complex component, in HUVECs were retrieved from the ChIP-Atlas platform (GEO: GSM2486813). CTCF ChIP-seq datasets for HUVEC were retrieved from ENCODE (ID: ENCSR000ALA-ENCFF334OZC).

##### HBMEC data

DNase-seq and CTCF ChIP-seq datasets for human brain microvascular endothelial cells (HBMEC) were retrieved from ENCODE (GEO: GSE169792 and GEO: GSM749743).

##### RNA-seq data

RNA sequencing (RNA-seq) data for HUVECs were retrieved from the ENCODE Project (GEO:GSE187975).

#### Chromatin interaction model

A hypothetical model of chromatin interactions between the *ABO* and *ADAMTS13* loci was drawn with BioRender using integrated datasets.

### Cell culture

HUVECs (American Type Culture Collection, catalog no. CRL-1730) were maintained with Dulbecco’s modified Eagle’s medium (DMEM) (Gibco, catalog no. 12100-046), supplemented with 5% fetal bovine serum (FBS) (Cultilab, catalog no. F063) and 1% penicillin (100 U/mL)/streptomycin (0.1 mg/mL) (Gibco, catalog no. 15140122) for luciferase assay. For CRISPR activation (CRISPRa), HUVECs were maintained with DMEM, supplemented with 8% FBS and 1% penicillin/streptomycin. The cells were maintained at 37°C in an incubator with 5% CO_2_.

### Cloning and luciferase assay

For the luciferase assay, four ∼200-bp sequences were synthesized (Table S2), including two sequences containing the rs657152 variant (one with allele A and the other with allele C) and two sequences containing the rs505922 variant (one with allele C and the other with allele T). The synthesized sequences were cloned into the pGL3-Promoter vector (Promega, catalog no. E1761) using the BglII and XhoI restriction sites.

Transfection was performed using Lipofectamine 3000 (Invitrogen, catalog no. L3000001). Cells were seeded in 24-well plates at a density of 40,000 cells per well in technical replicates (*n* = 3). Cells were allowed to adhere and grow for 22 h. Two hours prior to transfection, the medium was replaced. All wells were co-transfected with the pRL-SV40 plasmid (Promega, catalog no. E2231) to normalize luminescence readings.

Cells were transfected with 100 ng plasmid containing one of the sequences of interest (rs657152-A, rs657152-C, rs505922-C, or rs505922-T) and 1 ng pRL-SV40 plasmid. As a negative control, cells were transfected with 100 ng of the empty pGL3-Promoter vector and co-transfected with 1 ng pRL-SV40 plasmid. Luciferase expression was measured 24 h post-transfection.

### Cloning and chromatin editing (CRISPRa)

The single-guide RNA (sgRNA) sequences (Table S3) were designed using CRISPOR and cloned into the KpnI and EcoRI sites of the lentiGuide-Puro plasmid, a gift from Feng Zhang (Addgene plasmid no. 52963). For each target region containing the rs657152 or rs505922 variants, three distinct sgRNAs were used for CRISPRa.

HUVECs were plated in 24-well plates at a density of 40,000 cells per well in technical replicates (*n* = 3). After 22 h, the culture medium without antibiotics was replaced. Two hours later, transfections were performed using Lipofectamine 3000.

For each target region, 100 ng of each of the three distinct sgRNAs cloned into lentiGuide-Puro were co-transfected with 200 ng activation plasmid dCas9-VP64_GFP, also a gift from Feng Zhang (Addgene plasmid no. 61422). As a negative control, 300 ng empty lentiGuide-Puro vector was co-transfected with 200 ng dCas9-VP64_GFP activation plasmid.

Twenty-four hours after transfection, the medium was replaced and 40 h after the start of transfection, total RNA was extracted. Reverse transcription was performed using the SuperScript IV VILO Master Mix (Invitrogen, catalog no. 11756050). Quantitative PCR was conducted with TaqMan Fast Advanced Master Mix (Applied Biosystems, catalog no. 4444556) and TaqMan probes for ADAMTS13 (Applied Biosystems, catalog no. Hs00260148_m1) and glyceraldehyde 3-phosphate dehydrogenase (Applied Biosystems, catalog no. Hs02786624_g1).

### Allele-specific motif analysis

We used Tomtom (MEME Suite version 5.5.3) to test allele-specific motif disruption within ±10 bp of each variant, prioritizing statistical ranking and transcription factors expressed in basal HUVECs.

### Statistical analysis

The statistical analyses were conducted using the software RStudio. Statistical significance was assessed using an unpaired Student’s *t* test, with ∗*p* < 0.05. The graphs regarding statistical analyses were generated using the R package ggplot2 (version 3.5.1).

## Results

Analysis of the variants from the GWAS Catalog revealed that the *ABO* locus on chromosome 9 harbors the highest density of variants associated with VWF levels (Figure 1B; Table S1), followed by the *VWF* gene locus on chromosome 12.

To explore the possibility that non-coding variants associated with VWF levels at the *ABO* locus could engage in chromatin interactions with genes involved in coagulation and thrombosis, we analyzed a total of 45 annotated genes within a 500-kb region upstream and downstream of *ABO*. This analysis identified A Disintegrin and Metalloproteinase (ADAM) with thrombospondin motifs 13 (*ADAMTS13*), located approximately 147 kb from *ABO*, as a potential candidate for further investigation due to its functional connection between phenotypes strongly associated with ABO-linked non-coding variants, plasma levels of VWF, and thrombotic risk.

**Figure 2:**
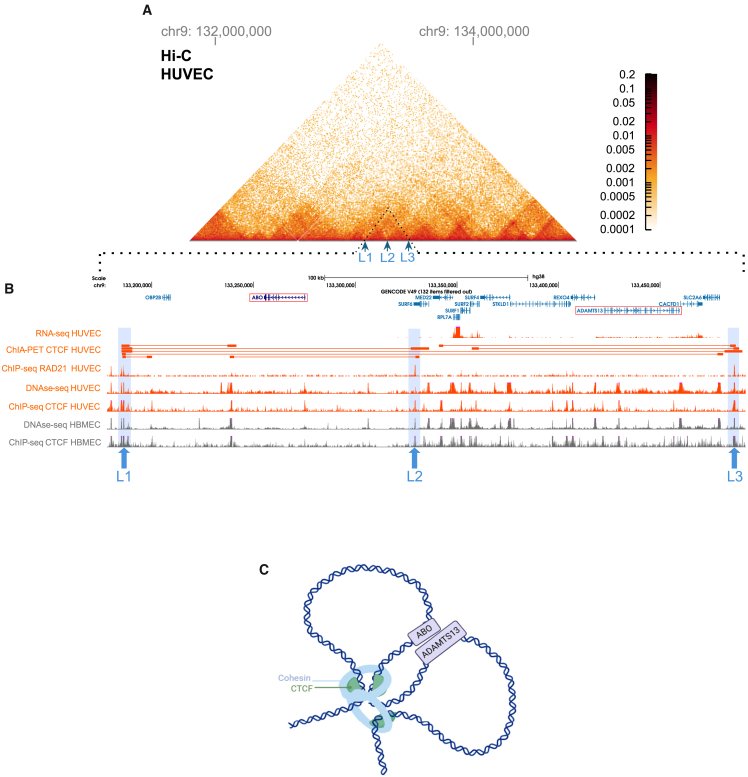
Chromatin interactions and regulatory landscape at the ABO and ADAMTS13 loci in endothelial cells (A) Hi-C data from HUVECs showing chromatin interactions within a segment of chromosome 9, encompassing the *ABO* and *ADAMTS13* loci. Three loop boundaries (L1, L2, L3) are identified, indicating regions anchoring chromatin loops. (B) Detailed epigenomic landscape of the *ABO* and *ADAMTS13* loci, corresponding to the region amplified in (C). Data from multiple assays are shown. RNA-seq data from HUVEC showing gene expression levels at the *ABO* and *ADAMTS13* loci. *ADAMTS13* is expressed in HUVECs, whereas *ABO* shows no detectable expression in this cell type. ChIA-PET (CTCF): identifies CTCF-binding at L1, L2, and L3, suggesting these sites demarcate loop boundaries. DNase-seq: reveals accessible chromatin regions aligning with the loop anchors. ChIP-seq for RAD21 (cohesin): confirms cohesin enrichment at L1, L2, and L3. ChIP-seq data for HBMECs identify CTCF binding sites, while DNase-seq reveals accessible chromatin regions at L1, L2, and L3. (C) Drawn chromatin interaction model of the *ABO* and *ADAMTS13* loci based on epigenomic and chromatin conformation data integrating Hi-C, ChIA-PET, DNase-seq, and ChIP-seq data from HUVEC, created with BioRender.com.

Using Hi-C data from the 4D Nucleome project, we mapped the chromatin interactions of the *ABO* and *ADAMTS13* regions from endothelial cells (HUVECs). This analysis revealed that *ABO* and *ADAMTS13* are topologically associated (Figure 2A). From the GWAS Catalog, at the *ABO* locus, we have identified a total of 26 variants related to VWF levels, 29 variants related to VTE, and 43 variants related to COVID-19 (Table S1, A1–A3). Genome-wide, a total of 308 variants associated with VWF levels were identified (Table S1, A4). After manual inspection, *ABO* non-coding variants linked to VWF levels, such as rs657152 (NC_000009.12:g.133263862A>C), rs9411377 (NC_000009.12:g.133269992A>C), rs660340 (NC_000009.12:g.133272138G>A), and rs505922 (NC_000009.12:g.133273813C>T), were identified in chromatin regions interacting with *ADAMTS13* (Figure S1). These four variants were deemed strong distal regulatory candidates, overlapping chromatin regions with topological interactions connecting the *ABO* and *ADAMTS13* loci. GWASs identified associations between rs657152 and VWF levels in varying scenarios,^9^^,^^12^ which justifies its inclusion despite being absent from the GWAS Catalog search associated with VWF in the supplemental information.

We next investigated chromatin interactions through ChIA-PET for CTCF, obtained from HUVECs (Figure 2B). ChIA-PET combines ChIP with proximity ligation and sequencing to detect protein-mediated chromatin interactions, which, together with cohesin, demarcate loop boundaries (L1, L2, and L3, as shown in Figures 2A and 2B). Additionally, ChIP-seq data for RAD21 were incorporated from HUVECs via the ChIP-Atlas platform (Figure 2B). Based on epigenomic and chromatin conformation data, we drew a chromatin interaction model of the *ABO* and *ADAMTS13* loci (Figure 2C).

**Figure 3:**
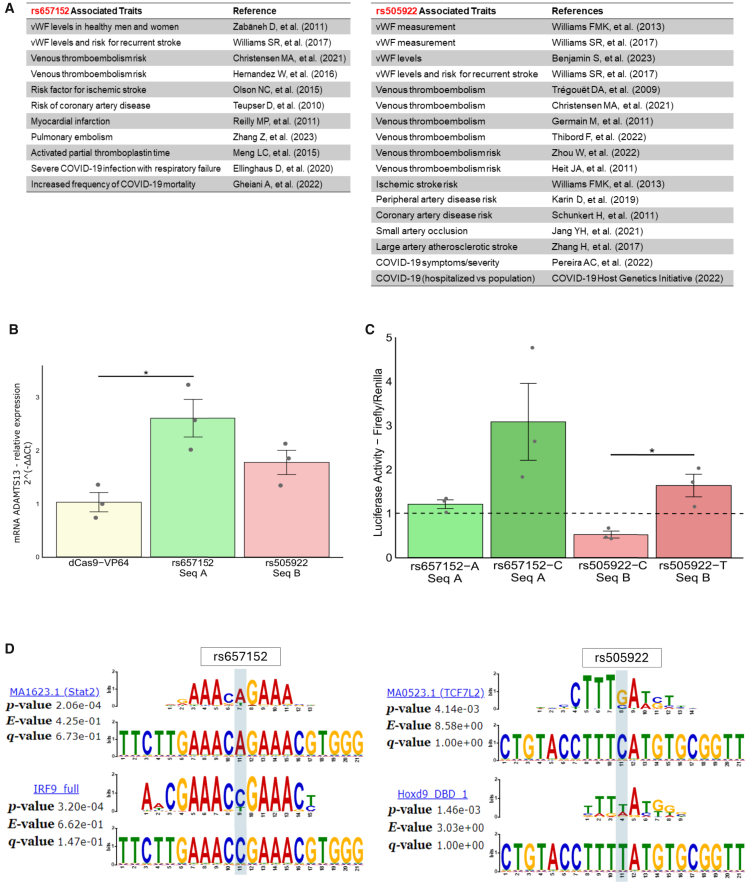
Functional testing and motif evaluation of non-coding variants at the ABO locus (A) Tables summarizing phenotypes previously associated with the non-coding variants rs657152 and rs505922 at the *ABO* locus. (B) Results of CRISPRa activation assays targeting the regions containing rs657152 and rs505922 in HUVECs. Three sgRNAs were used to activate transcription at each locus. The region harboring rs657152 demonstrated a significant increase in *ADAMTS13* expression (*p* < 0.05), supporting its role as a *cis*-regulatory element. In contrast, targeting the rs505922 region showed no significant activation. Data are represented as average fold change relative to control, with each point being a technical replicate (*n* = 3). (C) Results of luciferase reporter assays for the genomic regions containing rs657152 and rs505922. For each variant, constructs containing either the risk or non-risk allele were tested in HUVECs to evaluate allele-specific transcriptional activity. The assay showed that the risk allele of rs505922 significantly reduced luciferase activity compared to the non-risk allele (*p* < 0.05). In contrast, no statistically significant difference was observed between the alleles of rs657152. Data are represented as average luciferase activity relative to control, normalized with Renilla luciferase activity, with each point being a technical replicate (*n* = 3) for each variant. (D) Motif analysis for the genomic regions containing rs657152 and rs505922. The *in silico* analysis demonstrated allele-specific transcription factor binding motifs for both variants. Substitution of the risk and non-risk alleles resulted in distinct motif profiles.

To expand our findings we initiated functional testing with CRISPRa to determine whether regions within *ABO* that contact *ADAMTS13* could influence its transcription. For this assay, HUVECs were transfected with plasmids containing dCas9-VP64 and sgRNAs designed to target genomic regions of interest. Each region, harboring the variants rs657152 and rs505922 (Figure 3A; Table S4), was tested in separate experiments using three sgRNAs per variant. Notably, within the region containing the variant rs657152, there is another variant, rs147622835, which has no reported association with *ABO* or related phenotypes. CRISPRa targeting the region of rs657152 resulted in a significant increase in *ADAMTS13* transcription (Figure 3B), with consistent results across independent experiments.

To evaluate whether different alleles of rs657152 and rs505922 influence transcriptional activation, we performed luciferase reporter assays by transfecting HUVECs with constructs containing either allele variant of rs657152 or rs505922. For each variant, two constructs were generated: one with the risk allele and another with the non-risk (reference) allele. Luciferase assay showed that for the rs505922, the risk allele (C) significantly reduced transcriptional activation compared to the T allele (Figure 3C).

Allele-specific motif analysis for SNPs rs657152 and rs505922 revealed distinct transcription factor binding preferences. For rs657152, the A allele aligns with the motif for Stat2 (MA1623.1), while the C allele corresponds to IRF9 (IRF9_full). For rs505922, the G allele is associated with TCF7L2 (MA0523.1), whereas the A allele aligns with Hoxd9 (Hoxd9_DBD_1) (Figure 3D).

## Discussion

The analysis of the distribution of genetic variants associated with VWF levels in the genome showed that most *ABO*-associated variants reside in non-coding regions, suggesting they could act as *cis*-regulatory elements influencing gene expression, if they are functionally relevant. *Cis*-regulatory elements are essential for controlling gene expression. Variants within these regions may modulate transcriptional activity by disrupting transcription factor binding, chromatin architecture, or enhancer-promoter interactions, which are increasingly recognized as potential contributors to the pathogenesis of complex diseases.^21^

After assessing regions near the *ABO* for potential interactions via chromatin conformation, *ADAMTS13* emerged as a particularly interesting gene. Known to encode a protease with an established role in VWF physiology, its identification prompted further investigation. ADAMTS13 is mainly synthesized by hepatic stellate and endothelial cells. Given the ubiquitous distribution of endothelial cells, they likely represent a significant source of this protease.^22^ ADAMTS13 is the principal molecular regulator of the platelet-binding activity of VWF, cleaving highly procoagulant ultralarge VWF multimers into smaller, less active forms. Dysregulation of the ADAMTS13-VWF axis can result in hemostatic abnormalities, manifesting as bleeding or thrombosis. In addition, ADAMTS13 is implicated in thrombotic thrombocytopenic purpura (MIM: 274150) and von Willebrand disease (MIM: 193400).^23^

Chromatin conformation capture data revealed that *ABO* and *ADAMTS13* are topologically associated in endothelial cells. Furthermore, data from HBMECs revealed consistent chromatin accessibility and CTCF enrichment (Figure 2B) at three regions identified as potential loop-forming elements linking *ABO* and *ADAMTS13*. Having observed the topological association between non-coding *ABO* variants and the *ADAMTS13* gene, we sought to determine whether this proximity could influence *ADAMTS13* transcription. To this end, we employed CRISPRa, a technique that utilizes catalytically inactive dCas9 fused to VP64, a potent transcriptional activator domain. The selection of the rs657152 and rs505922 variants for these functional assays was informed by substantial prior evidence linking these SNPs to phenotypes including VWF levels, VTE, and COVID-19 (Figure 3A). CRISPRa assays demonstrated that the rs657152 region can enhance ADAMTS13 transcription, suggesting that the spatial proximity of *ABO* and *ADAMTS13* may facilitate regulatory interactions influencing *ADAMTS13* gene expression.

A study investigating gastric cancer cells reported that the deletion of a downstream region of the *ABO* gene altered the expression of nearby genes, including a reduction in *ADAMTS13* transcription.^24^ Although this finding was incidental to the primary focus of that study, which examined the regulation of the Odorant Binding Protein 2B gene, it highlights a broader context in which variations within the *ABO* locus may influence transcriptional activity of *ADAMTS13*.

The rs505922-C allele demonstrated a capacity to inhibit transcriptional activation in endothelial cells. Since allele substitutions can influence transcription factor recruitment by reshaping the regulatory landscape, this variant could have functional implications beyond transcriptional regulation. The non-coding SNPs rs657152 and rs505922 are in linkage disequilibrium with the O-determining deletion rs8176719 at the *ABO* locus and are frequently used as genetic proxies for the ABO blood group in large-scale association studies.^13^^,^^25^^,^^26^^,^^27^ Furthermore, even under standardized conditions, luciferase assays for the rs657152-C allele showed variability, which is expected for this method. Although additional replicates could improve detection of subtle effects, the current replication level was considered sufficient for qualitative interpretation. Our findings further reveal that rs505922 is topologically linked to *ADAMTS13*, suggesting that if functionally relevant, it could influence not only ABO-related traits, as extensively reported in the literature, but also ADAMTS13-related phenotypes. Supporting this hypothesis, individuals with blood group O exhibit approximately 10% higher ADAMTS13 levels and 35% lower VWF levels compared with those with blood groups A, B, or AB.^23^ Several studies indicate that the *ABO* locus harbors more than one independent association signal. While rs505922, in strong linkage disequilibrium with the O-determining deletion, is thought to reflect ABO blood group effects on VWF glycosylation, our findings suggest that non-coding variants such as rs657152 appear to involve *cis*-regulatory mechanisms of *ADAMTS13*. These mechanisms are not mutually exclusive and together could contribute to the variability in VWF levels.

One proposed mechanism reported in the literature to explain ABO-VWF associations involves post-translational glycosylation of the VWF protein by ABO blood group antigens. The addition of A or B antigens to N-linked glycans on VWF may influence its susceptibility to ADAMTS13-mediated proteolysis and its clearance by hepatocytes and macrophages, potentially contributing to lower VWF levels observed in individuals with blood group O.^17^^,^^28^^,^^29^^,^^30^

While our study demonstrates that *ABO* non-coding variants associated with VWF levels are topologically linked to *ADAMTS13*, it does not establish a direct correlation between these variants and plasma ADAMTS13 levels. Prior studies identified SNPs upstream of *ABO* associated with ADAMTS13 levels, but not with ABO blood group phenotypes, suggesting alternative regulatory mechanisms.^31^

We observed divergent activity patterns for rs657152 and rs505922, consistent with distinct regulatory layers assayed. CRISPRa reflects the endogenous chromatin environment, capturing 3D organization and epigenetic accessibility, whereas luciferase assays test isolated sequence fragments in episomal settings. This suggests that rs657152 acts through chromatin-dependent mechanisms, while rs505922 mediates sequence-intrinsic effects detectable in minimal reporter constructs.

Our motif analyses suggested potential STAT2 and IRF9 disruptions at rs657152 (Figure 3D). These proteins usually act together as part of the ISGF3 complex in canonical type I interferon signaling. To our knowledge, there is no direct evidence linking STAT2 or IRF9 activity to the regulation of VWF or ADAMTS13 in endothelial cells. Thus, these results should be viewed cautiously and may indicate a context-dependent regulatory mechanism not yet characterized.

Our chromatin conformation analyses provide evidence that non-coding variants in *ABO*, associated with VWF levels, VTE risk, and COVID-19 severity, are in spatial proximity to *ADAMTS13* in endothelial cells. While these findings highlight a potential topological organization linking these variants to a key coagulation-related gene, further studies are required to clarify the functional implications of this chromatin interaction. Additionally, a better understanding of how genetic variation and 3D genome architecture contribute to coagulation phenotypes, particularly in the regulation of plasma and cellular ADAMTS13, is needed.

## Data and code availability

This study did not generate/analyze datasets or code.

## Acknowledgments

The authors thank the 10.13039/501100001807São Paulo Research Foundation for funding this research (#20/13240-3). D.V.E. and H.F.d.S.S. are supported by 10.13039/501100002322CAPES (Coordination for the Improvement of Higher Education Personnel). The authors thank Dr. Nadav Ahituv, professor at the University of California, San Francisco, as well as the members of his laboratory, for valuable discussions that contributed to this study.

## Author contributions

D.V.E. and H.F.d.S.S. conducted experimental investigations, analyzed the data, and participated in manuscript preparation. M.R.M. developed the hypothesis, conceptualized the study, secured funding, supervised the research, conducted experimental investigations, analyzed the data, and contributed to manuscript preparation.

## Declaration of interests

The authors declare no competing interests.

## Declaration of generative AI and AI-assisted technologies in the writing process

During the preparation of this work, the authors used OpenAI’s ChatGPT to improve the clarity and readability of the English language and to generate Figure 1B. After using this tool, the authors reviewed and edited the content as needed and take full responsibility for the content of the publication.

## Web resources

4D Nucleome Project, https://4dnucleome.org
Addgene, https://addgene.org
BioRender, https://biorender.com
ChIP-Atlas, https://chip-atlas.org
CRISPOR, https://crispor.gi.ucsc.edu
ENCODE Project, https://www.encodeproject.org
ggplot2, https://ggplot2.tidyverse.org/
GWAS Catalog, https://www.ebi.ac.uk/gwas
MEME Suite, https://meme-suite.org/meme
Online Mendelian Inheritance in Man, http://www.omim.org
University of California, Santa Cruz Genome Browser, https://genome.ucsc.edu

## Supplemental information

Document S1. Figure S1 and Tables S2–S4

Table S1. Genetic variants The genetic variants are associated with the following: A1. VWF: Variants linked to plasma von Willebrand Factor (VWF) levels at the ABO locus. A2. VTE: Variants associated with venous thromboembolism (VTE) risk at the ABO locus. A3. COVID-19: Variants related to susceptibility and severity of COVID-19 at the ABO locus. A4. Genetic variants associated with von Willebrand Factor (VWF) levels. This table summarizes data from genome-wide association studies (GWASs) identifying variants linked to VWF levels.

Document S2. Article plus supplemental information
