# Supplementary material for: A non-coding ABO regulatory variant associatedwith VWF levels, thrombosis risk, and COVID-19 severity is topologically linked to ADAMTS13 in endothelial cells
Source: HGG Adv. 2025 Nov 27;7(1):100550. doi: 10.1016/j.xhgg.2025.100550 (PMC12765440; doi:10.1016/j.xhgg.2025.100550)
Supplement: Document S1. Figure S1 and Tables S2–S4 [file mmc1.pdf]

HGGA, Volume 7

Supplemental information

A non-coding ABO regulatory variant associatedwith VWF levels, thrombosis risk, and COVID-19 severity is topologically linked to ADAMTS13 in endothelial cells

Douglas Victorino Esposito, Hellen Ferreira de Souza Sobrinho, and Marcelo Rocha Marques

Figure S1

In situ Hi-C data from HUVECs highlighting chromatin contacts between four genetic variants (rs657152, rs9411377, rs660340, rs505922) at *ABO*, and *ADAMTS13*.

Data source: 4DNESHFBC56P - 4D Nucleome. A 3D map of the human genome at kilobase resolution reveals principles of chromatin looping. Rao et al., Cell 2014.

a) **rs657152**

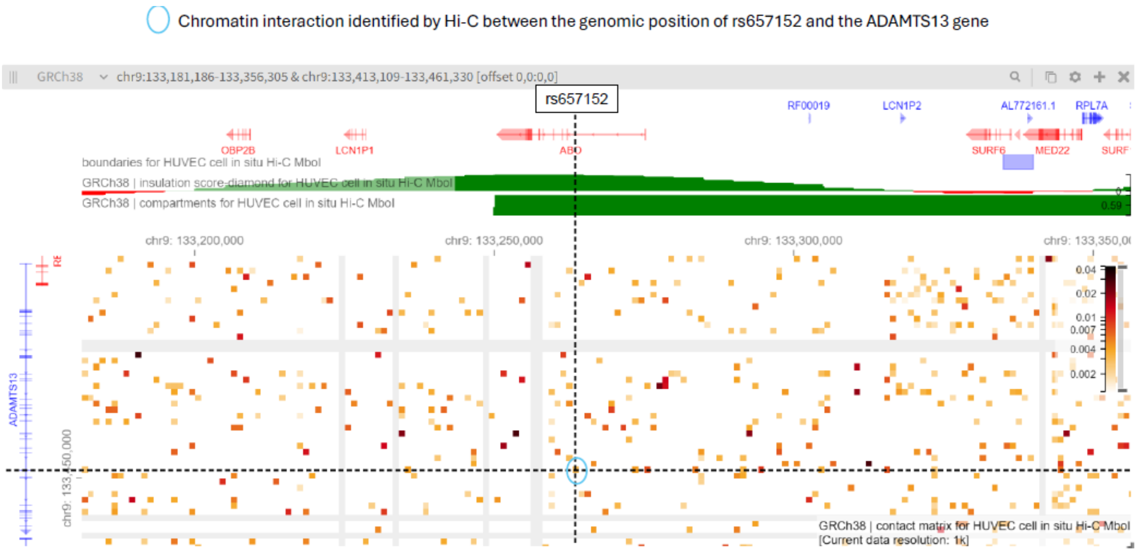

b) rs9411377

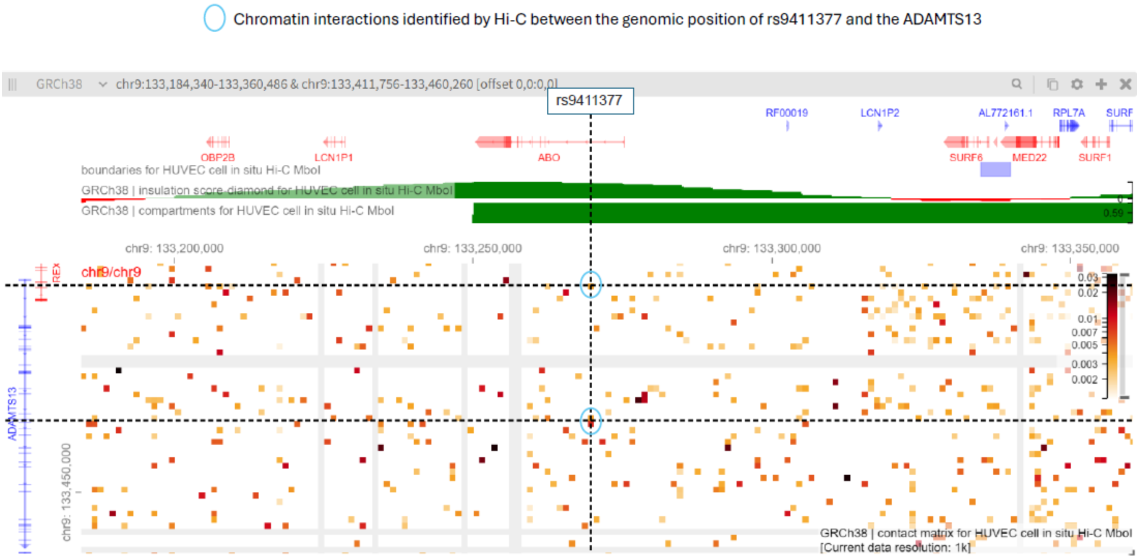

c) rs660340

Chromatin interactions identified by Hi-C between the genomic position of rs660340 and the ADAMTS13

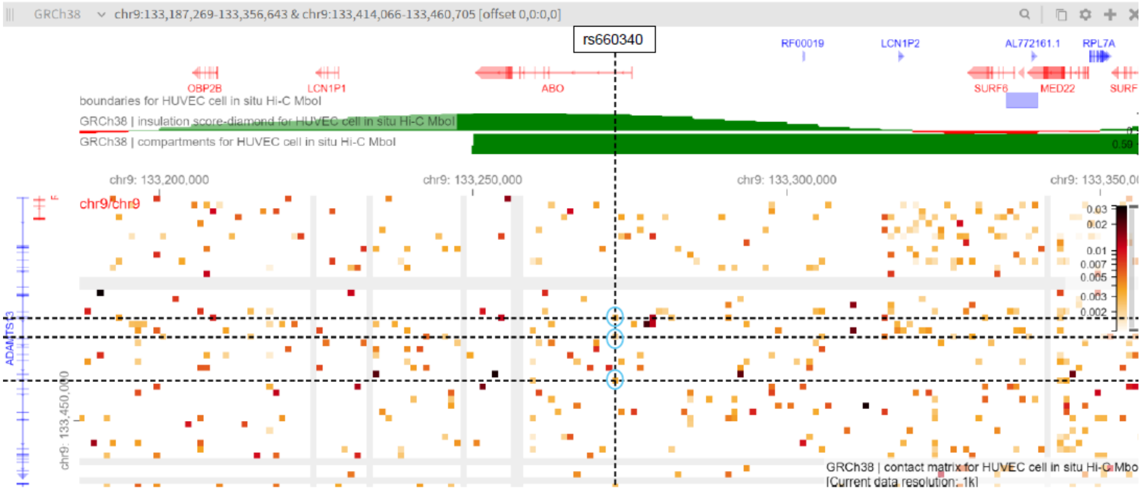

d) rs505922

Chromatin interactions identified by Hi-C between the genomic position of rs505922 and the ADAMTS13

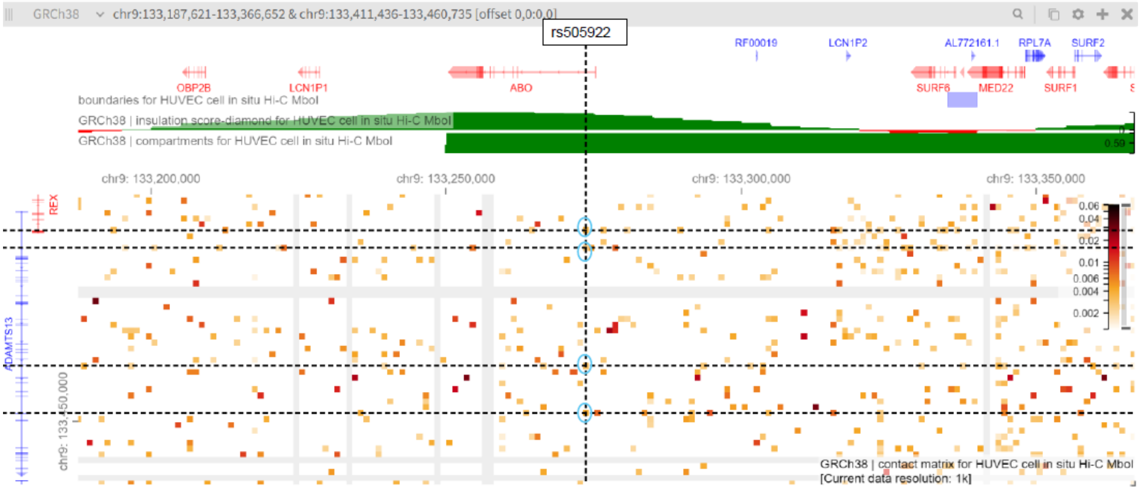

Table S2: Genomic sequences and risk alleles used for functional assays.

Luciferase Cloned Sequences

Set	Variant	Genomic Position (hg38)
SeqA	rs657152-A (risk allele)	chr9:133,263,834-133,264,033
	rs657152-C	
SeqB	rs505922-C (risk allele)	chr9:133,273,562-133,273,821
	rs505922-T	

Table S3: sgRNAs targeting regions around rs657152 and rs505922 used for CRISPRa experiments.

CRISPRa Cloned gRNAs

Set	Name	Genomic Position (hg38)	Sequence
SeqA	sgA1	chr9:133,263,850-133,263,869	GCTTCTTGAAACAGAAACGT
	sgA2	chr9:133,263,736-133,263,755	TTGAAAGGGACTGATTTCGG
	sgA3	chr9:133,263,808-133,263,827	GGTCACAGGACTTAGAAAGG
SeqB	sgB1	chr9:133,273,736-133,273,755	TGGGGCAGGACAGACTCCTG
	sgB2	chr9:133,273,802-133,273,821	CCGCACATGAAAGGTACAGC
	sgB3	chr9:133,273,755-133,273,774	ATGAACACAGCTGCCACCCC

Table S4: References of the studies cited in Figure 3A.

rs657152

Reference	PMID
Zabaneh D, et al. (2011)	PMID: 21534939
Williams SR, et al. (2017)	PMID: 28495826
Christensen MA, et al. (2021)	PMID: 34777790
Hernandez W, et al. (2016)	PMID: 26888256
Olson NC, et al. (2015)	PMID: 26286125
Teupser D, et al. (2010)	PMID: 20529992
Reilly MP, et al. (2011)	PMID: 21239051
Zhang Z, et al. (2023)	PMID: 37076872
Weng LC, et al. (2015)	PMID: 25552651
Ellinghaus D, et al. (2020)	PMID: 32558485
Gheinari, et al. (2022)	PMID: 36419842

rs505922

Reference	PMID
Williams FMK, et al. (2013)	PMID: 23381943

Williams SR, et al. (2017)	PMID: 28495826
Benjamin B Sun, et al. (2023)	PMID: 37794186
Williams SR, et al. (2017)	PMID: 28495826
Trégouët DA, et al. (2009)	PMID: 19278955
Christensen MA, et al. (2021)	PMID: 34777790
Germain M, et al. (2011)	PMID: 21980494
Thibord F, et al. (2022)	PMID: 36154123
Zhou W, et al. (2022)	PMID: 36777996
Heit JA, et al. (2011)	PMID: 21463476
Williams FMK, et al. (2013)	PMID: 23381943
Klarin D, et al. (2019)	PMID: 31285632
Schunkert H, et al. (2011)	PMID: 21378990
Jiang YY, et al. (2021)	PMID: 33053422
Zhang H, et al. (2017)	PMID: 26924317
Pereira AC, et al. (2022)	PMID: 35368071
COVID-19 Host Genetics Initiative. (2022)	PMID: 35922517
